# The efficacy of sonographic measurement of inferior vena cava diameter as an estimate of central venous pressure

**DOI:** 10.1186/s12947-016-0076-1

**Published:** 2016-08-20

**Authors:** William Ciozda, Ilan Kedan, Devin W. Kehl, Raymond Zimmer, Raj Khandwalla, Asher Kimchi

**Affiliations:** 1 David Geffen School of Medicine, University of California, Los Angeles, CA USA; 2 Cedars-Sinai Heart Institute, Cedars-Sinai Medical Group, Beverly Hills, CA USA; 3 Cedars-Sinai Heart Institute, Los Angeles, CA USA

**Keywords:** Inferior vena cava, Central venous pressure, Right atrial pressure, Ultrasound

## Abstract

**Background:**

Central venous pressure (CVP) and right atrial pressure (RAP) are important parameters in the complete hemodynamic assessment of a patient. Sonographic measurement of the inferior vena cava (IVC) diameter is a non-invasive method of estimating these parameters, but there are limited data summarizing its diagnostic accuracy across multiple studies. We performed a comprehensive review of the existing literature to examine the diagnostic accuracy and clinical utility of sonographic measurement of IVC diameter as a method for assessing CVP and RAP.

**Methods:**

We performed a systematic search using PubMed of clinical studies comparing sonographic evaluation of IVC diameter and collapsibility against gold standard measurements of CVP and RAP. We included clinical studies that were performed in adults, used current imaging techniques, and were published in English.

**Results:**

Twenty one clinical studies were identified that compared sonographic assessment of IVC diameter with CVP and RAP and met all inclusion criteria. Despite substantial heterogeneity in measurement techniques and patient populations, most studies demonstrated moderate strength correlations between measurements of IVC diameter and collapsibility and CVP or RAP, but more favorable diagnostic accuracy using pre-specified cut points. Findings were inconsistent among mechanically ventilated patients, except in the absence of positive end-expiratory pressure.

**Conclusion:**

Sonographic measurement of IVC diameter and collapsibility is a valid method of estimating CVP and RAP. Given the ease, safety, and availability of this non-invasive technique, broader adoption and application of this method in clinical settings is warranted.

## Background

Measurement of central venous pressure (CVP) is a critical component of the complete hemodynamic assessment of a patient. CVP is considered equivalent to right atrial pressure (RAP) when the vena cava is continuous with the right atrium [1]. Central venous catheterization is the gold standard measurement of CVP and RAP [1]. However, widespread and routine use of this invasive procedure are limited by the risk of complications, including infection, catheter-induced thrombosis, and arrhythmias [2]. Therefore, noninvasive techniques to estimate CVP play a crucial role in promoting more widespread CVP evaluation in clinical practice [1].

The inferior vena cava (IVC) is a compliant vessel whose size and shape vary with changes in CVP and intravascular volume [1]. Therefore, sonographic measurement of the IVC represents an effective and noninvasive method of estimating CVP [3, 4]. However, several factors may affect IVC size. Under normal physiologic conditions, IVC diameter decreases and venous return increases during inspiration due to negative intrathoracic pressure and positive intra-abdominal pressure [5]. IVC diameter also decreases during ventricular systole [1]. Additionally, patient position may affect IVC diameter, as the diameter is smallest when the patient is in the left lateral position and largest when the patient is in the right lateral position [6]. Awareness of these variables is critical to the accurate collection and interpretation of sonographic IVC measurements.

Early standards for sonographic IVC assessment called for measurements of the maximum IVC diameter (IVCmax) and the minimum IVC diameter (IVCmin) during the respiratory cycle [5]. An IVC collapsibility index (IVCCI), which correlates with RAP and CVP, can be calculated with the following formula: (IVCmax − IVCmin)/IVCmax [3]. Guidelines on echocardiographic chamber quantification published in 2015 from the American Society of Echocardiography recommend that the maximum IVC diameter be measured from the subcostal view with the IVC displayed along its long axis [7]. The diameter should be measured immediately caudal to the junction of the hepatic vein with the IVC and approximately 1–2 cm caudal to the junction of the IVC and the ostium of the right atrium [7]. Although prior iterations of these guidelines have recommended these measurements be performed with the patient in the left lateral position [8] and at the end of expiration [9], the supine position is now recommended, and currently no specific recommendation is made as to the phase of the respiratory cycle during which to perform the measurement. Measurement of the IVCCI with a brief sniff is also recommended in combination with IVC diameter in order to estimate CVP as normal (0–5 mmHg), intermediate (5–10 mmHg), or high (10–20 mmHg) [7].

A number of clinical studies have evaluated IVC diameter measurements as a method to estimate CVP and RAP, but the overall reliability and accuracy of this technique has not been systemically compared across multiple studies. We provide a comprehensive review of the existing literature to examine the reliability and accuracy of sonographic measurement of IVC diameter as a method for assessing CVP and RAP.

## Methods

We performed a systematic literature search in PubMed with search terms “ultrasound of inferior vena cava and central venous pressure” and “ultrasound of inferior vena cava and right atrial pressure” to identify clinical studies that compared ultrasound measurement of IVC diameter and collapsibility against gold standard measurements of CVP and RAP. Only clinical research studies among adults that pertained to our objective, used up-to-date imaging techniques, and were published in English were included in our review. For each study, we collected data on study design, patient population, and major findings, including the correlation between invasive pressure measurements and IVC measurement parameters.

## Results

**Fig. 1:**
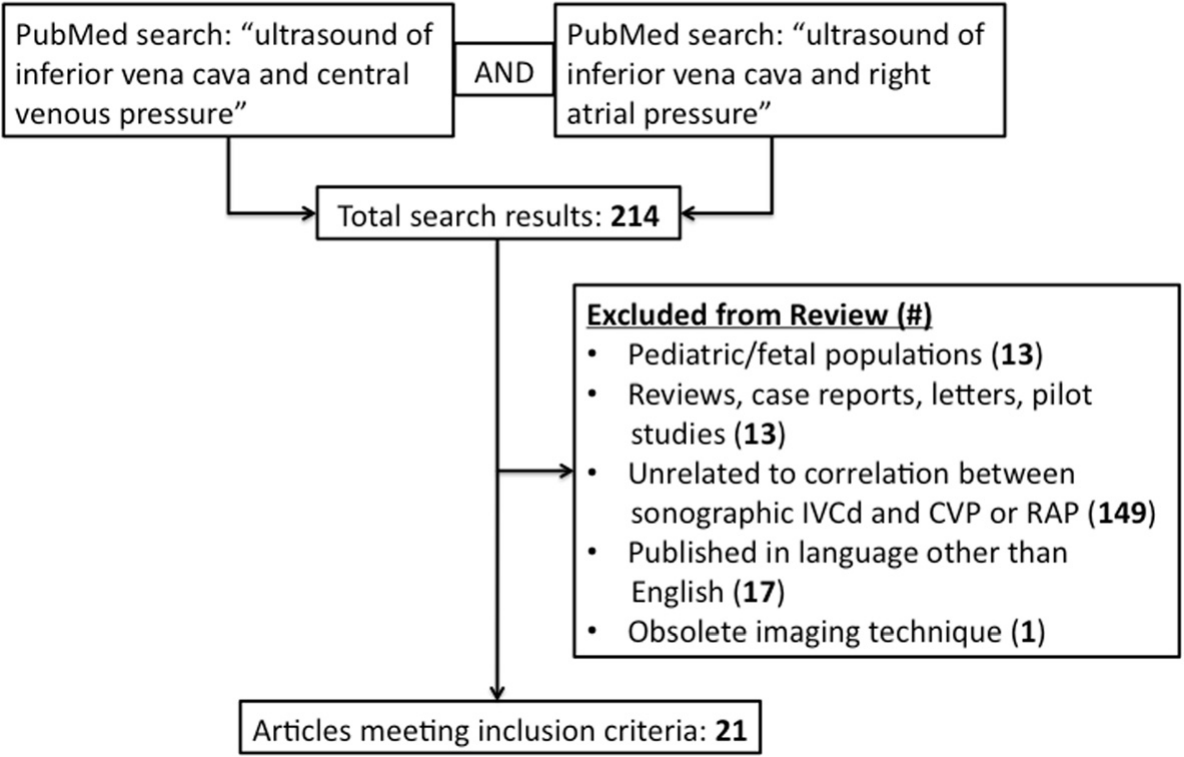
Study selection flowchart showing the structure of the PubMed search and exclusion criteria

Our literature search returned 214 journal articles, of which we excluded 13 studies that were performed in pediatric/fetal populations, 13 that were not clinical research studies, 149 that were unrelated to our objective, 17 that were not published in English, and 1 that used an obsolete imaging technique (Fig. 1). A total of 21 studies that examined the correlation between sonographic measurements of IVC diameter and CVP or RAP were reviewed. The sample sizes across studies ranged from 22 to 175 patients, with a total of 1,430 patients across all studies combined.

In all studies, IVC measurements were taken from the supine position, using the subcostal view (Table 1). However, there were substantial differences between the studies in terms of the patient population, use of mechanical ventilation with positive end-expiratory pressure (PEEP), mean CVP or RAP, and method of IVC diameter measurement. Furthermore, the specific IVC measurement parameters varied across studies. These included IVCmax, maximum IVC diameter at end-expiration (IVCe), maximum IVC diameter at end-inspiration (IVCi), and IVCCI. All studies reported correlations between IVC measurements and direct invasive measurements of CVP or RAP.

**Table 1:** Methodology and sample descriptions for studies examining IVC diameter as an estimate of CVP or RAP

Authors	Year	N	Patient population	Percent on mechanical ventilation	PEEP (cm H_2_O)	Gold standard comparison	Mean CVP or RAP	Patient position	Sonographer	Other notes
Taniguchi et al. [19]	2015	90	Elective right heart catheterization	0 %	n/a	RAP	8 mmHg	Supine	Sonographer	9 % post-heart transplant
Sobczyk et al. [24]	2015	50	Elective cardiac surgery	100 %	4.5	CVP	6.7 mmHg	Supine	Sonographer	
Tsutsui et al. [22]	2014	75	Decompensated heart failure	0 %	n/a	RAP	13 mmHg	Supine	Sonographer	
Zhang et al. [21]	2014	72	Gastrointestinal surgery with hypovolemia	0 %	n/a	CVP	3 mmHg	Supine	Sonographer	Repeated after fluids
Citilcioglu et al. [25]	2014	45	ER patients with invasive monitor	24 %	5	CVP	7.7 mmHg (8.7 mmHg with mechanical ventilation)	Supine	^a^	
Prekker et al. [26]	2013	65	Medical ICU patients	0 %	n/a	CVP	7 mmHg (median)	Supine	MD in IM, ICU, ER	
De Lorenzo et al. [27]	2012	65	ER and ICU patients with critical illness	43 %	^a^	CVP	10.4 cmH_2_O	Supine	ER MD or RN, ICU MD	Subxiphoid views in 57 patients
Patel et al. [16]	2011	36	Decompensated heart failure	0 %	n/a	RAP	11 mmHg	Supine	Sonographer	20 % moderate TR 8 % severe TR 25 % AF
Yildirimturk et al. [15]	2011	39	Rheumatic mitral stenosis	0 %	n/a	RAP	9.7 mmHg	Supine	Sonographer	44 % in AF
Nagdev et al. [28]	2010	73	Critical ER patients with central catheter	19 %	^a^	CVP	10.5 mmHg	Supine	ER MD	
Schefold et al. [29]	2010	30	Severe sepsis, septic shock	100 %	12	CVP	15 cmH_2_O	Supine	ICU MD	
Arthur et al. [10]	2009	95	Elective cardiac surgery	100 %	0; ventilator turned off	CVP	14.5 mmHg	Supine	Anesthesia MD	TEE only
Lorsomradee et al. [11]	2007	70	Elective cardiac surgery	100 %	0, 5, 10	CVP	11, 12, 14 mmHg	Supine	Anesthesia MD	TEE only
Brennan et al. [18]	2007	102	Elective right heart catheterization	0 %	n/a	RAP	7 mmHg	Supine	Sonographer	9 % in AF 30 % post-heart transplant
Ommen et al. [30]	2000	71	Cardiac catheterization lab	0 %	n/a	RAP	^a^	Supine	^a^	
Nagueh et al. [31]	1996	35	Elective right heart catheterization or critical illness	34 %	^a^	RAP	9 mmHg	Supine	Sonographer	
Jue et al. [12]	1992	49	ICU or CCU	100 %	^a^	RAP	^a^	^a^	^a^	
Kircher et al. [3]	1990	83	Cardiac catheterization lab	0 %	n/a	RAP	11 mmHg	^a^	Sonographer	
Simonson et al. [32]	1988	27	Awake patients with pulmonary arterial catheters	0 %	n/a	RAP	^a^	Supine	^a^	
Moreno et al. [17]	1984	175	80 healthy volunteers; 95 with cardiac abnormalities; 65 with right heart catheterization	0 %	n/a	RAP	>7 mmHg in 35/65 patients	^a^	Sonographer	17 % in AF
Mintz et al. [20]	1981	50	Elective right heart catheterization	0 %	n/a	RAP	^a^	Supine	Sonographer	BSA adjusted

^a^data not available

The majority of studies reported statistically significant positive correlations between sonographic measurements of IVC diameter and CVP or RAP. All studies that examined the relationship between ultrasound measurements of maximum IVC diameter and CVP or RAP across the entire respiratory cycle, at end-expiration, or at end-inspiration reported positive correlations (Table 2). All studies that provided *p*-values for the correlation reported statistically significant results at α = 0.05.

**Table 2:** Published correlations between ultrasound measurements of maximum IVC and CVP or RAP among spontaneously ventilating patients

Measurement time-point	Study	N	Mean CVP or RAP	Correlation coefficient	*p*-value	Comments
Entire respiratory cycle	Taniguchi et al., [19]	90	8.0	0.67	<0.05	
	Yildirimturk et al., [15]	22	9.7	0.62	<0.005	Patients with normal sinus rhythm only; When all patients (*n* = 39) included, *r* = 0.51 (*p* < 0.005).
	Patel et al., [16]	36	11.0	0.56	<0.001	
	Brennan et al., [18]	91	7.0	0.50	^a^	
	Ommen et al., [30]	71	^a^	0.86	<0.001	
	Moreno et al., [17]	65	^a^	0.40	^a^	
End-expiration	Citilcioglu et al., [25]	34	7.7	^a^	0.002	
	Tsutsui et al., [22]	71	13.0	0.40	<0.0001	
	Zhang et al., [21]	40	3.0	0.59	<0.01	
	Prekker et al., [26]	65	7.0	0.76	<0.05	Study reports median CVP/RAP
	De Lorenzo et al., [27]	57	10.4	0.47	<0.05	43 % of patient received mechanical ventilation
	Nagdev et al., [28]	73	10.5	0.66	<0.05	19 % of patients received mechanical ventilation
	Nagueh et al., [31]	23	9.0	0.40	0.05	
	Kircher et al., [3]	83	^a^	0.48	^a^	
	Mintz et al., [20]	50	^a^	0.72	<0.001	
End-inspiration	Citilcioglu et al., [25]	34	7.7	^a^	0.001	
	Tsutsui et al., [22]	71	13.0	0.49	<0.0001	minimum diameter during sniff
	De Lorenzo et al., [27]	29	10.4	0.69	<0.05	extrapolated from data provided
	Nagdev et al., [28]	73	10.5	0.78	<0.05	
	Kircher et al., [3]	83	^a^	0.71	^a^	
	Simonson et al., [32]	27	^a^	0.56	^a^	minimum diameter during inspiration
	Simonson et al., [32]	27	^a^	0.35	^a^	

^a^data not available

Multiple studies also reported statistically significant negative correlations between IVCCI and CVP or RAP (Table 3). Although some studies measured IVCCI during passive inspiration while others measured it during forceful inspiration (sniff), neither method had a consistently stronger correlation with invasive CVP or RAP measurements.

**Table 3:** Published correlations between ultrasound measurements of IVC collapsibility index and CVP or RAP in spontaneously ventilating patients

Type of inspiration	Study	N	Mean CVP or RAP	Correlation coefficient	*p*-value	Comments
Passive	Taniguchi et al., [19]	90	8.0	−0.57	<0.05	Study reports median CVP/RAP
	Zhang et al., [21]	72	3.0	−0.27	0.017	
	Prekker et al., [26]	65	7.0	−0.40	<0.05	Study reports median CVP/RAP
	Yildirimturk et al., [15]	22	9.7	−0.49	<0.05	
	Nagdev et al., [28]	73	10.5	−0.74	<0.05	
	Brennan et al., [18]	91	7.0	−0.50	^a^	
	Kircher et al., [3]	83	^a^	−0.75	^a^	
	Moreno et al., [17]	65	^a^	−0.71	^a^	
Sniff	Taniguchi et al., [19]	90	8.0	−0.63	<0.05	Study reports median CVP/RAP
	Tsutsui et al., [22]	71	13.0	−0.41	<0.0001	
	Patel et al., [16]	34	11.0	−0.49	0.006	
	Brennan et al., [18]	91	7.0	−0.54	^a^	
	Nagueh et al., [31]	23	9.0	−0.76	<0.001	

^a^data not available

Several studies have also identified threshold levels of IVC size and collapsibility by which to estimate CVP or RAP (Table 4). Although the specific threshold values for IVC size, IVCCI, CVP, and RAP varied slightly across studies, the diagnostic accuracy of IVC measurements parameters was generally high, with the C-statistic ranging from 0.76–0.91 for IVC diameter and 0.66–0.93 for IVCCI.

**Table 4:** Diagnostic performance characteristics of IVC size parameters for the prediction of CVP or RAP among spontaneously ventilating patients

Parameter	Study	N	Parameter cut-point	Outcome	Sensitivity	Specificity	Positive predictive value	Negative predictive value	Area under ROC curve
IVCmax	Taniguchi et al., [19]	90	≥2.0 cm	RAP ≥ 10 mmHg	^b^	^b^	^b^	^b^	0.83^a^
	Prekker et al., [26]	65	<2.0 cm	CVP < 10 mmHg	0.85	0.81	0.87	0.78	0.91^a^
	Patel et al., [16]	40	≥2.0 cm	RAP > 10 mmHg	0.89	0.67	^b^	^b^	0.76^a^
	Brennan et al., [18]	46	>2.0 cm	RAP > 10 mmHg	0.73	0.85	0.62	0.90	0.76^a^
	Moreno et al., [17]	65	>2.3 cm	RAP > 7 mmHg	0.40	0.97	0.93	0.58	^b^
IVCCI (passive)	Taniguchi et al., [19]	90	<25 %	RAP ≥ 10 mmHg	^b^	^b^	^b^	^b^	0.79^a^
	Prekker et al., [26]	65	>50 %	CVP < 10 mmHg	0.47	0.77	0.75	0.50	0.66^a^
	Patel et al., [16]	40	<40 %	RAP > 10 mmHg	^b^	^b^	^b^	^b^	0.67
	Nagdev et al., [28]	73	>50 %	CVP < 8 mmHg	0.91	0.94	0.87	0.96	0.93^a^
	Brennan et al., [18]	46	<20 %	RAP > 10 mmHg	0.73	0.82	0.57	0.90	0.93^a^
	Moreno et al., [17]	65	<40 %	RAP > 7 mmHg	0.91	0.9	0.91	0.90	^b^
IVCCI (sniff)	Taniguchi et al., [19]	90	<50 %	RAP ≥ 10 mmHg	^b^	^b^	^b^	^b^	0.83^a^
	Brennan et al., [18]	46	<40 %	RAP > 10 mmHg	0.73	0.84	0.62	0.90	0.91^a^
	Nagueh et al., [31]	23	<50 %	RAP > 8 mmHg	0.72	0.76	^b^	^b^	^b^
	Kircher et al., [3]	83	<50 %	RAP > 10 mmHg	0.87	1.00	1.00	0.92	^b^
IVCmax and IVCCI (passive)	Patel et al., [16]	40	≥2.0 cm and <40 %	RAP > 15 mmHg	0.86	0.73	^b^	^b^	^b^
	Patel et al., [16]	40	≥2.0 cm and <40 %	RAP > 10 mmHg	0.60	0.83	^b^	^b^	^b^

^a^Statistically significant at alpha = 0.05

^b^data not available

The reported correlations between IVC dimension and CVP in mechanically ventilated patients are generally weak and inconsistent across studies (Table 5). Approximately half of the studies in this patient population did not detect a statistically significant correlation. Correlations that did reach statistical significance in mechanically ventilated patients were mostly weak to moderate in strength. The notable exceptions were the two studies in which no PEEP was used during ventilation, with correlation coefficients of 0.80 and 0.86 for the relationship between IVCe and CVP or RAP [10, 11].

**Table 5:** Published correlations between ultrasound measurements of IVC diameter and CVP or RAP among mechanically ventilated patients

Parameter	Study	N	Mean CVP or RAP (mmHg)	PEEP (cm H_2_O)	Correlation coefficient	*p*-value
IVCmax	Sobczyk et al., [24]	50	6.7	4.5	0.18	0.034
	Nagueh et al., [31]	12	9.0	*	0.4	NS
IVCe	Citilcioglu et al., [25]	11	8.7	5.0	*	NS
	Schefold et al., [29]	30	15.0^a^	12.0	0.56	0.001
	Arthur et al., [10]	95	14.5	0	0.86	<0.0001
	Lorsomradee et al., [11]	70	10.0	0	0.8	<0.001
	Lorsomradee et al., [11]	70	14.0	10.0	0.27	NS
	Jue et al., [12]	49	*	*	0.58	0.001
	Citilcioglu et al., [25]	11	8.7	5.0	*	NS
	De Lorenzo et al., [27]	29^b^	10.4	*	0.26	NS
IVCi	Schefold et al., [29]	30	15.0^a^	12.0	0.51	0.004
	Sobczyk et al., [24]	50	6.7	4.5	−0.19	0.008
	Nagueh et al., [31]	12	9.0	*	0.24	NS
IVCCI	Jue et al., [12]	49	*	*	0.13	NS
	Nagueh et al., [31]	12	9.0	*	0.24	NS
	Jue et al., [12]	49	*	*	0.13	NS

^a^cm H_2_O

^b^extrapolated from data provided

*data not available

## Discussion

Overall, these findings support the use of sonographic measurements of IVC diameter to estimate CVP or RAP in spontaneously ventilating patients. Positive correlations were consistently reported between IVC size and CVP or RAP, and negative correlations were consistently reported between IVCCI and CVP or RAP. Although the correlations were generally only moderately strong, the diagnostic performance of pre-specified cut-points was superior, and justifies the current guideline recommendations for estimation of right-sided filling pressure. Importantly, there was substantial heterogeneity across the studies reviewed in the timing of IVC size measurement with respect to the respiratory cycle. Although IVCe has previously been recommended as the preferred IVC parameter by which to estimate CVP or RAP [9], the strength of the correlations between CVP and IVCe, IVCi, and IVCmax were similar. This is reflected in the most recent guidelines by the American Society of Echocardiography, which do not specify an optimal phase of the respiratory cycle during which to measure the maximal IVC diameter.

The correlations between IVC dimension and CVP in mechanically ventilated patients were generally weak and inconsistently observed. Furthermore, the use and magnitude of PEEP varied greatly across studies of mechanically ventilated patients. Positive pressure ventilation leads to increased intrathoracic pressure, decreased systemic venous return, and increased volume of venous blood in the IVC. The dimension and distensibility of the IVC is consequently affected. Therefore, the use of IVC measurements to estimate RAP in mechanically ventilated patients is usually unreliable. Accordingly, 2015 guidelines from the American Society of Echocardiography recommend against their routine application in this setting [7]. However, in the study by Jue and colleagues, despite only a modest correlation between RAP and IVC dimension, these authors did find that an IVC diameter of 1.2 cm or less had 100 % specificity for a right atrial pressure less than 10mmHg, albeit with poor sensitivity (25 %) [12]. Therefore, a small IVC in the setting of mechanical ventilation may still point toward the absence of elevated RAP. In addition, the correlation of IVCe and RAP may still be valid in the absence of PEEP.

There are a few other notable circumstances in which IVC diameter may not correlate with CVP or RAP. First, the IVC may be dilated in young elite athletes with normal RAP, particularly swimmers. One study showed a mean IVC diameter of 2.3 cm in elite athletes compared to 1.3 cm in control subjects [13]. In addition, young patients with vasovagal syncope but no other cardiac history have been found to have increased IVC size as compared to controls, suggestive that venous pooling in young healthy patients may increase IVC size independent of any increase in atrial pressure [14]. Furthermore, the CVP or RAP can be highly dynamic, such as in the setting of severe tricuspid regurgitation, and IVC size should not be relied upon as an accurate estimate in this setting. Finally, invasive measurement of the CVP or RAP is also subject to its own pitfalls and measurement error. These can be numerous, and with accurate measurements being dependent on proper catheter function and pressure transduction, leveling, and tip positioning.

There was substantial heterogeneity across the reviewed studies with respect to patient population. Importantly, the validity of IVC measurements for CVP or RAP estimation may not be equivalent in all patient subgroups. One population with limited data includes patients with atrial fibrillation, in whom venous inflow Doppler pattern is altered due to the loss of the atrial relaxation wave. Only four studies included patients with atrial fibrillation [15–18], with such patients comprising a minority of the population in each study. None of these studies assessed the validity of these measurements specifically among patients with atrial fibrillation. Further investigation is warranted in these patients. Patients with a history of heart transplantation, included in several of the studies described in this review, represent another subgroup worthy of additional research. Whether mechanical disruption of the IVC with caval anastomosis affects its performance as an indicator of RAP has not been described, but could be clinically important.

There are only limited data suggesting an effect of body size on IVC diameter [19], and to support indexing IVC diameter to body surface area (BSA) for the estimation of RAP. A study from 1981 observed a weak correlation of indexed IVC diameter with RAP [20], and a more contemporary study showed no improvement in diagnostic performance of IVC measurements as an estimate of RAP with indexing to BSA [18]. A study from 2015 showed no improvement in diagnostic performance after indexing IVC diameter to BSA, but found a significantly lower optimal cut point for IVC diameter in the estimation of RAP among patients with small BSA [19]. Guideline recommendations currently do not recommend indexing IVC size to BSA.

One valuable application of non-invasive sonographic estimation of CVP may lie in serial measurements. IVC diameter has been shown to increase after fluid resuscitation and in association with concomitant increases in CVP [21]. Additionally, although the precision of IVC-derived estimation of CVP may be reduced in heart failure, serial assessment can be performed in patients with decompensated heart failure in order to guide management [22]. This simple parameter, easily measurable at the point of care, has been found to offer as much precision as more complex estimates involving more variables [22].

Simplification of IVC measurements could improve their standardization and application in clinical practice. In a pilot study, Martin et al. demonstrated success in training hospitalists to perform sonographic IVC measurements using an online module and a 1-day training session [23]. After the session, 8 of 10 hospitalists were able to accurately acquire and interpret IVC images in 5 of 5 patients and discern whether the IVCCI was greater than 50 % with 91 % accuracy. The success of this short training program not only exemplifies the ease of both IVC ultrasound training and use but also a model by which it could be applied more broadly.

## Conclusion

Ultrasound measurement of the IVC at the point of care provides insight into hemodynamics in a rapid and non-invasive manner and can impact clinical decision making. Assessment of CVP, historically requiring invasive intervention, can be performed non-invasively with reasonable accuracy in most, but not all, clinical settings. Additional validation of IVC measurements for estimation of CVP may be indicated in specific subgroups of patients. Measurement of the IVC with portable ultrasound devices as well as additional health care provider training may allow for expansion of filling pressure estimation as an extension of the routine bedside clinical examination of all patients.

## Abbreviations

AF, atrial fibrillation; BSA, body surface area; CCU, critical care unit; CVP, central venous pressure; ER, emergency room; ICU, intensive care unit; IM, internal medicine; IVC, inferior vena cava; IVCCI, inferior vena cava collapsibility index; IVCe, maximum diameter of the inferior vena cava at end-expiration; IVCi, maximum diameter of the inferior vena cava at end-inspiration; IVCmax, maximum diameter of the inferior vena cava across the respiratory cycle; MD, medical doctor; N, sample size; n/a, not applicable; PEEP, positive end-expiratory pressure; RAP, right atrial pressure; RN, registered nurse; ROC, receiver operating characteristic; TEE, transesophageal echocardiogram; TR, tricuspid regurgitation.
